# An animal model in sheep for biocompatibility testing of biomaterials in cancellous bones

**DOI:** 10.1186/1471-2474-7-67

**Published:** 2006-08-15

**Authors:** Katja MR Nuss, Joerg A Auer, Alois Boos, Brigitte von Rechenberg

**Affiliations:** 1 Musculoskeletal Research Unit (MSRU), Equine Hospital, Vetsuisse Faculty, Zurich, Switzerland; 2 Institute of Veterinary Anatomy, Vetsuisse Faculty, Zurich, Switzerland

## Abstract

**Background:**

The past years have seen the development of many synthetic bone replacements. To test their biocompatibility and ability for osseointegration, osseoinduction and -conduction requires their placement within bone preferably in an animal experiment of a higher species.

**Methods:**

A suitable experimental animal model in sheep with drill holes of 8 mm diameter and 13 mm depth within the proximal and distal humerus and femur for testing biocompatibility issues is introduced.

**Results:**

This present sheep model allows the placing of up to 8 different test materials within one animal and because of the standardization of the bone defect, routine evaluation by means of histomorphometry is easily conducted. This method was used successfully in 66 White Alpine Sheep. When the drill holes were correctly placed no complications such as spontaneous fractures were encountered.

**Conclusion:**

This experimental animal model serves an excellent basis for testing the biocompatibility of novel biomaterials to be used as bone replacement or new bone formation enhancing materials.

## Background

The use of resorbable and non- resorbable biomaterials, such as hydroxyapatite or tricalcium phosphate, as synthetic bone replacements is well established in orthopaedic, maxillofacial and dental surgery [1,2]. Although autologous cancellous bone is the material of choice for bone replacement and induction there are limitations in relation to its use, such as limited amount of material, additional surgical procedure, prolonged surgery and complications of wound healing at the donor site. Because of this there is continuous interest in the development of new synthetic materials [3-5].

The most important aspect in the development of new (biodegradable) biomaterials is the experimental and clinical testing for biocompatibility [6-8]. This is closely followed by the bioactivity, which relates to the resorption or integration of the implanted material into the surrounding bone, also called "osseointegration" and the ability to initiate and support the apposition and integration of the new bone, in particular in comparison with previously established materials [4].

Various animal species are used for these biocompatibility tests, such as the mouse [9], rat [10-18], guinea pig [19], rabbit [20-23], dog [24-28] goat [29] and sheep [30-32]. Furthermore, different implantation sites and methods have been used to examine biocompatibility issues. Among them are intra-peritoneal [13], subcutaneous [12,14,17,33], intraosseous such as in the mandible [19,27], femur [11,15,24,25]), tibia [18,20,21,23,26,30], cranial bone [28,31] and intramuscular [9,16,29] applications.

The use of sheep for orthopaedic research continues to increase. This is due to the similarities with humans in weight, bone and joint structure and bone regeneration [34-36]. Although rodents may be less expensive, they have different bone morphology. Another practical reason is that rodents often are too small in size to test degradable materials in bone especially in combination with internal fixation and fracture repair [37,38]. Furthermore, the influence of the different mechanical properties of bone from different species awaits clarification in relation to the outcome of these studies [39,40].

To contribute to the standardisation of testing new and biodegradable materials for use in orthopaedics, maxillofacial and dental surgery our research group has developed an animal model with sheep that allows the intraosseous implantation of 8 different samples per sheep in long bones [41,42]. This animal model facilitates testing inter- and intra-individual differences among different materials while at the same time reducing overall suffering of animals as well as necessary numbers to satisfy statistical requirements. It has been already successfully applied for several studies related to testing biodegradable materials [32] and to the knowledge of the authors has never been described before in the literature.

## Methods

### Instruments

**Figure 1:**
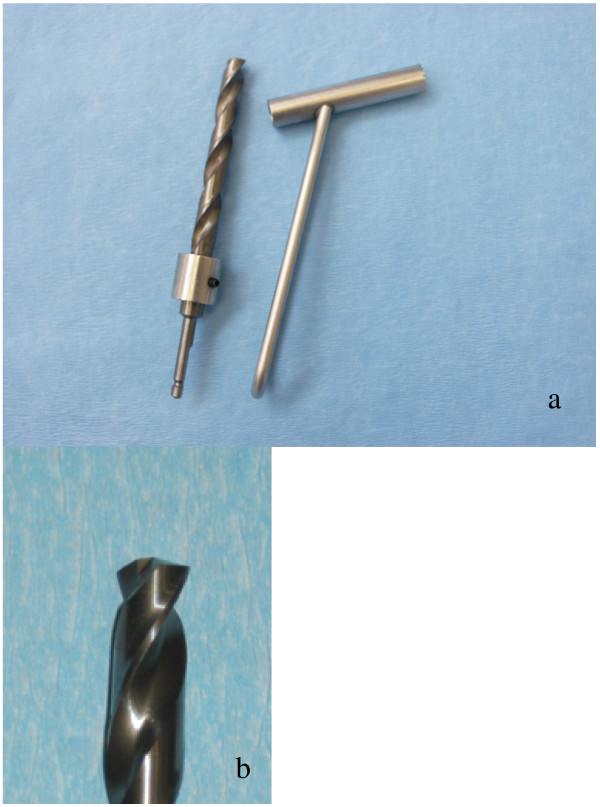
The photograph shows the 8 mm special depth-regulating bit and corresponding drill guide (la) and the close-up (lb) the flattened tip of the drill bit.

In addition to routine surgical instruments, Weitlaner and Gelpi-retractors, a periosteal elevator, pneumatic drill (Synthes, Waldenburg, Switzerland) with an 8 mm, slightly modified drill bit (KaVo INTrASurg 500^®^, KaVo Dental AG Biberach, Germany, modified by Synthes, Waldenburg, Switzerland) and a corresponding drill guide were used (Fig. 1). The drill bit was modified with a special depth-regulating device and the tip was flattened while still having good cutting characteristics.

### Biomaterials

Implanted were various cements (*β*-tricalciumphosphates, brushite, hydroxyapatite), hydrogels (fibrin-based, polyethylene glycol) and other resorbable or non-resorbable bone replacement materials as composites with or without growth factors such as parathyroid hormone (PTH_1–34_), bone morphogenic protein (BMP-2), transforming growth factor (TGF*β*) and insulin-like growth factor 1 (IGF-1).

### Experimental animals

**Figure 2:**
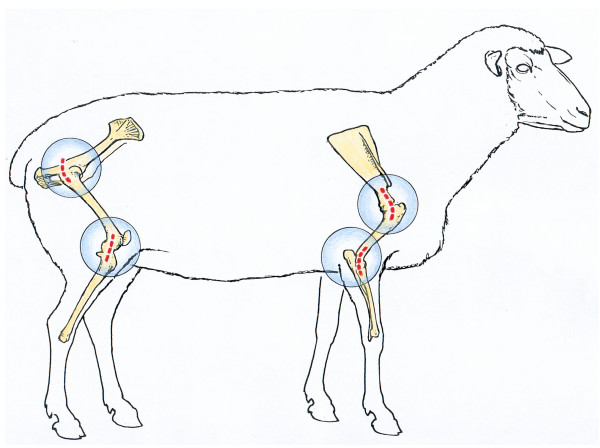
Layout of the approaches to the implantation sites: the dotted lines represent the sites of skin incisions.

Used were 70 healthy ewes, White Swiss Alpine Sheep, with an age of 2 to 5 years and a bodyweight of 53 to 80 kg. The implantation sites were the proximal part of the diaphysis and distal epiphysis of humerus and femur (Fig. 2). This provided a total of 8 implant sites per animal. All animal experiments were carried out according to the Swiss Laws of animal welfare and were approved by the Ethics Commission of the official veterinary authorities (Authorisation Numbers: 53/2000, 139/2000, 09/2002, 176/2003, 118/2004, 14/2005, 15/2005, 90/2005).

The experimental animals were kept in stalls prior to surgery. Food was withdrawn 36 hours, and water 6 hours prior to anaesthesia. A pre-anaesthetic examination was performed including haematology and a chemscreen consisting of liver enzymes, urea, creatinine, protein, albumin, sodium and potassium. After recovery of the animals from anaesthesia, they were initially kept in small groups in stables. After 10 days, the skin staples were removed and the animals were allowed to join the flock on the fields normally at 20 days after surgery. They were checked daily for lameness and additional clinical problems. Food and water were given ad libitum.

### Surgery

Animals were sedated with medetomidine (5 *μ*g/kg BW, Domitor™, Orion Pharma Animal Health, Finland) and anaesthesia was induced with ketamine (2 mg/kg BW, Narketan^® ^10, Chassot GmbH, Germany) in combination with diazepam (0.01 mg/kg BW, Valium^®^, Roche, Switzerland) and maintained with 0.8 Vol% isoflurane (Forene^®^, Abbot AG, Switzerland) in O_2 _and an infusion of Ringer's solution with 60 mg ketamine (Narketan™ 10, Chassot GmbH, Germany)/litre at a rate of 10 ml/kg BW/hour. The animals received as pre-and post-operative prophylaxis 30,000 IU penicillin/kg BW (Hoechst AG, Germany) and 6 mg gentamicin/kg BW (Streuli & Co AG, Switzerland) intravenously twice a day. In addition, they received subcutaneously 500 Units of equine tetanus serum as a single application (Tetanus Serum Veterinaria AG, Zurich, Switzerland).

Analgesia was maintained through injection of 0.01 mg buprenorphine/kg BW i.v. perioperatively and additionally 4 mg carprofen/kg BW i.v. (Rimadyl^®^, Pfizer Inc., NY, USA) postoperatively for 3 days. The area in the region of the incisions was clipped and disinfected in the standard manner.

The animals were placed in left or right lateral recumbency on the operating table with the limbs placed in a horizontal position. The approach to the bone was always from the lateral side (Fig. 2). It is important for the forelimb that the humerus is positioned parallel with the humeral condyles being at a 90° angle to the surgery table. Furthermore the limb should be slightly flexed to the position that the lateral epicondyle of the humeral condyle can be palpated easily directly under the skin. The hind limb should be also slightly flexed such that the lateral collateral ligament can be palpated through the skin, although a slight downward inclination of the hind limb may facilitate access to the proximal femur. Both, the fore- and hind limbs should be additionally supported from underneath so that they cannot be pushed down when drilling the hole but remain stable in the prepared plane.

With all approaches an attempt was made to keep the skin incisions and preparation of soft tissue down to the bone as small as possible (ca. 6–8 cm in length). To facilitate the further surgical approaches to all locations, the wounds were kept open by means of either a Weitlaner retractor alone or in combination with a Gelpi retractor that was usually placed perpendicular to the already placed Weitlaner retractor (proximal and distal femur). The periosteal elevator was used to prepare the location of the drill hole such that all overlying soft tissues were removed from the bone. This facilitated the placement of the drill guide and avoided the slippage of the drill bit initially before the drill had started to penetrate the cortical bone. The description of the four different surgical sites is given below. A total number of 560 drill holes were placed (n = 560 with 70 drill holes in each location). Immediately after drilling, the holes were filled with the various test materials. After filling the drill holes, the different wound layers as described in the approaches were closed separately with non-resorbable suture material (Vicryl 2/0, Johnson & Johnson, Brussels, Belgium) and the skin was stapled (Davis and Geck Appose ULCr, B. Braun Aesculap AG, Tuttlingen, Germany)

### Approaches

#### Proximal part of humeral diaphysis (Fig. 3)

**Figure 3:**
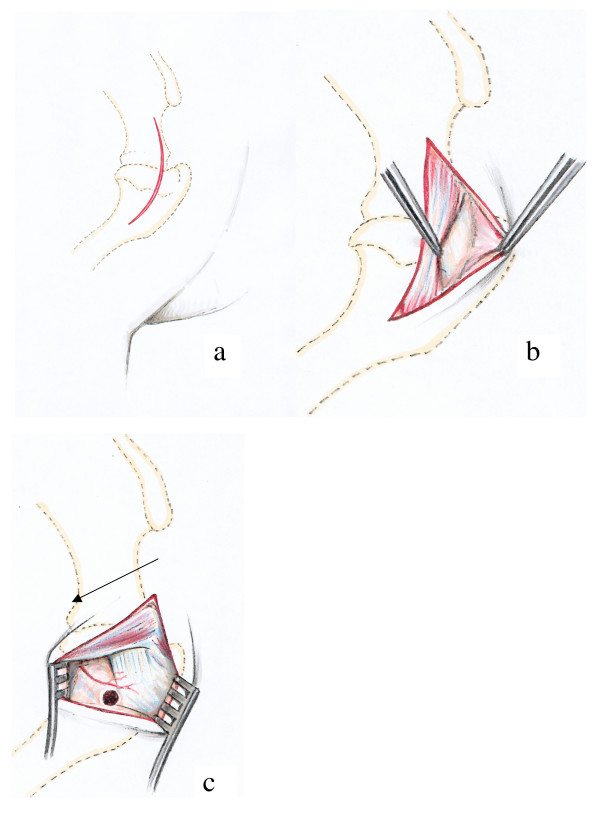
Surgical procedure at the proximal diaphysis of the humerus: The skin incision extends from the acromion to the distal end of the tuberculum majus (Fig. 3a). The acromial part of the deltoid muscle is dislocated caudally to expose the tendon of insertion of the infraspinatus muscle (Fig. 3b), where the hole is drilled caudally to the insertion point and distally to the unnamed vessel (arrow) (Fig. 3c).

The skin incision began at the level of the acromion and followed a slightly curved line over the shoulder joint to the middle of the proximal third of the humerus (Fig. 3a). After dissecting the subcutaneous fascia the acromial part of the deltoid muscle was visible and situated between the trapezius and the omotransversarius muscles. These muscles were then retracted caudally (Fig. 3b). Beneath appeared the tendon of insertion of the infraspinatus muscle. A hole was then drilled into the humerus caudally to this tendon and in the medial direction. A small vessel without a given name of less than 1 mm in diameter and crossing the bone directly above the insertion of the tendon served as a consistent landmark (Fig. 3c).

#### Distal epiphysis of the humerus (Fig. 4)

**Figure 4:**
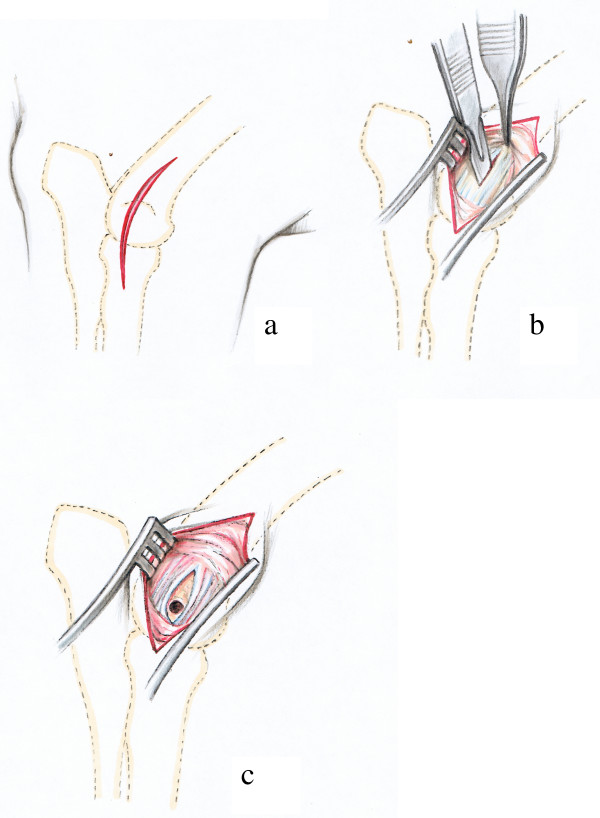
Surgical procedure at the distal epiphysis of the humerus: The skin incision is performed directly above the lateral epicondyle of the humerus (Fig. 4a). Then the proximal part of the Lig. collaterale laterale is incised with a scalpel following the direction of the fibres. The forceps tip is on the dorsal and prominent rim of a groove that gives origin to this ligament (Fig. 4b). The drill hole is situated directly in the centre of the groove between the fibres of the lateral collateral ligament (Fig. 4c).

A slightly curved skin incision was made laterally, directly over the lateral epicondyle of the humerus (Fig. 4a). After the dissecting of the subcutaneous and deep fasciae the lateral epicondyle giving origin to the lateral collateral ligament could be palpated. An incision was made in the ligament paralleling the direction of its fibres (Fig. 4b). The drill guide was passed through the opening and the drill hole situated exactly in the centre of the groove and the lateral humeral condyle (Fig. 4c).

#### Proximal part of the femoral diaphysis (Fig. 5)

**Figure 5:**
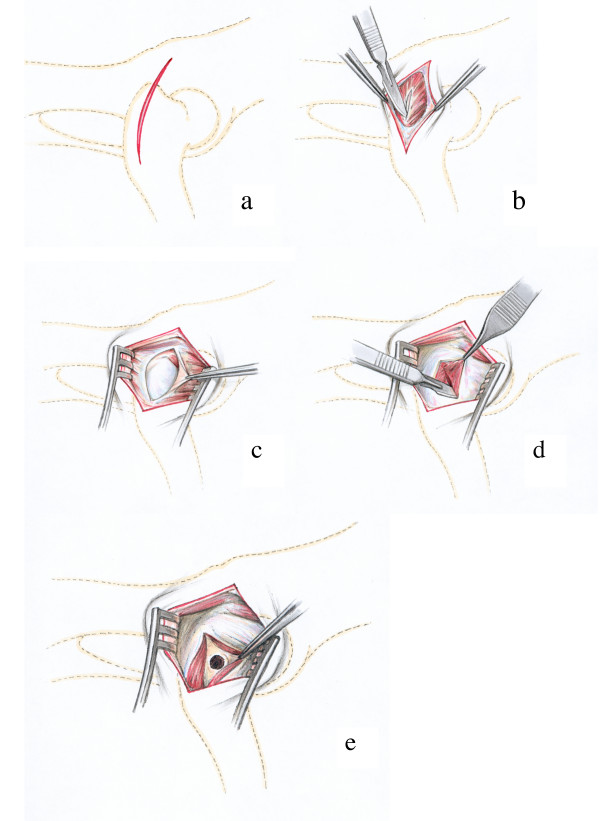
Surgical procedure at the proximal part of the femoral diaphysis: The skin incision is situated directly above the greater trochanter (Fig. 5a). The incision in the deep layer of the fascia lata reveales the medial gluteal muscle that is separated longitudinally (Fig. 5b) to expose the lateral of the quadriceps muscle (Fig. 5c). The lateral vastus is split longitudinally (Fig. 5d) and the final drill hole is inserted in the centre at the base and 1.5 cm distally to the tip of the greater trochanter (Fig. 5e).

The skin incision was made directly above the easily palpable greater trochanter of the femur (Fig. 5a). The medial gluteal muscle appeared immediately after the incision of the subcutaneous fascia. The muscle was penetrated in a longitudinal direction using a blunt incision (Fig. 5b). The underlying fascia lata was opened with a scalpel blade exposing the lateral vastus of the quadriceps muscle (Fig. 5c). The access to the bone was then made directly beneath the greater trochanter through a longitudinal incision of the lateral vastus of the quadriceps muscle (Fig. 5d). The drill guide was placed perpendicular to the bone axis and firmly pressed to the bone to avoid slippage during drilling. It was located 1.5 cm below the tip at the base and in the centre of the greater trochanter (Fig. 5e).

#### Distal epiphysis of the femur (Fig. 6)

**Figure 6:**
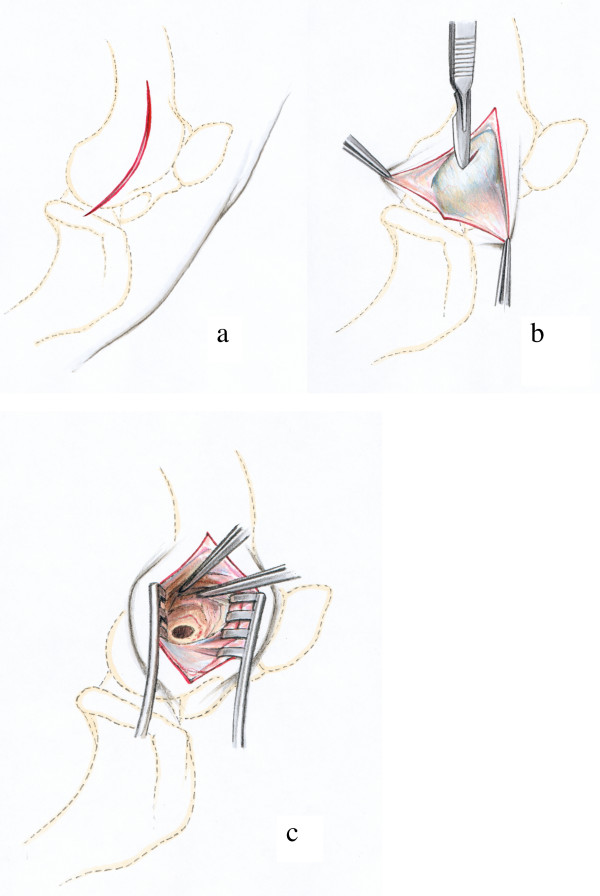
Surgical procedure at the distal epiphysis of the femur: The skin incision extends between the lateral femoral condyle and the tibial tuberosity of the tibia (Fig. 6a). The incision in the aponeurosis of the biceps femoris muscle (Fig. 6b) is made perpendicular to the muscle fibres of the biceps femoris muscle and the drill hole is inserted just proximal to the origin of the lateral collateral ligament of the stifle joint (Fig. 6c).

Palpable bone landmarks are the patella, the tibial tuberosity and the lateral condyles of the femur. The skin incision was performed in the triangle between the patella and the tibial tuberosity on the one and the lateral condyles of femur and tibia on the other side (Fig. 6a). With the splitting of the subcutaneous fascia the aponeurosis of the biceps muscle was visible. The muscle fascia was opened with a small (ca. 2 cm) incision perpendicular to the muscle fibres (Fig. 6b) and the underlying muscle tissue was bluntly dissected down to the lateral condyle of the femur. The hole was drilled closely to the proximal origin of the lateral collateral ligament of the stifle joint (Fig 6c).

**Figure 7:**
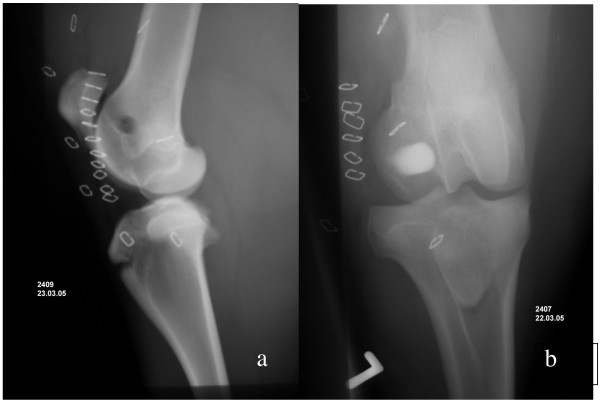
Postoperative lateromedial (Fig. 7a) and craniocaudal (Fig. 7b) radiograph of the distal part of the left femur after the injection of two specific, but different biomaterials. While bone cement (Fig. 7b) reveals immediate radiodensity, a biomaterial based on hydrogel and without radiopacifier cannot be visualized immediately after surgery. If bone formation takes place over time, radiodensity will increase gradually.

After surgery the exact locations of the material-filled holes could be determined using radiographs (Figs. 7a and 7b). Due to the different densities of the overlying soft tissues (pelvis and proximal humerus versus elbow and stifle joint), each radiograph had to be taken separately amounting to 8 radiographs per animal. If radiographic follow-ups had to be obtained at several time intervals, each time the sheep had to be subjected to a short anaesthesia (Sedation with medetomidine (5 *μ*g/kg BW, Domitor^®^, Orion Pharma Animal Health, Finland) and induction with ketamine (2 mg/kg BW, Narketan^® ^10, Chassot GmbH, Germany) in combination with diazepam (0.01 mg/kg BW, Valium^®^, Roche, Switzerland) and maintained with propofol (2–4 mg/kg BW, Disoprivan^® ^Astra Zeneca, Switzerland) to assure appropriate quality of the radiographs.

### Evaluation of samples

**Figure 8:**
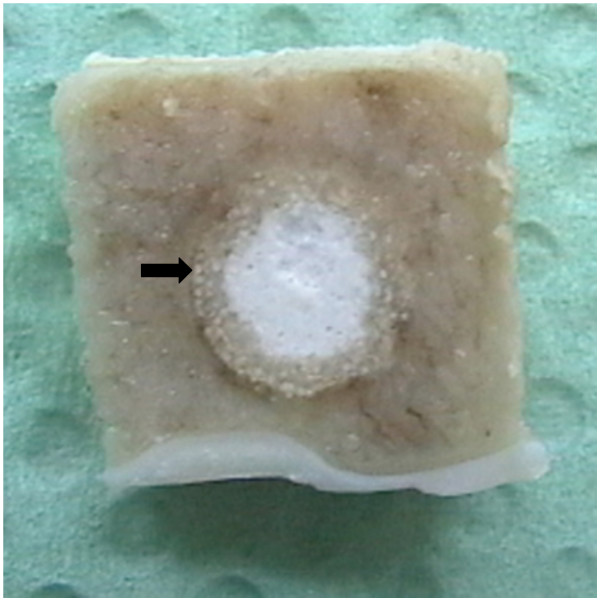
Preparation of the bone blocks after sacrifice: Note the rim of adjacent bone (arrow) around the original bone defect filled with biomaterial.

**Figure 9:**
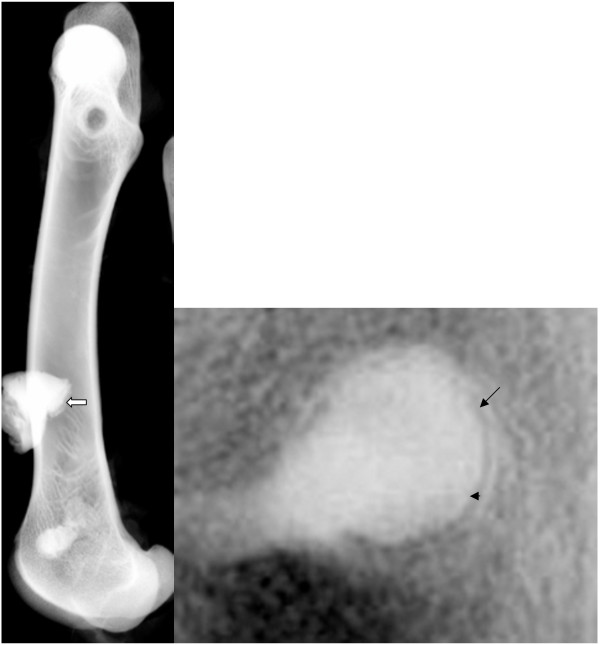
Radiograph taken with the faxitron machine: The technique, where the entire bone or bone samples already cut in blocks are radiographed with the faxitron machine, allows the assessment of bony changes, such as bone resorption (arrow head) or bone sclerosis (arrow) as a means for biocompatibility of the inserted biomaterials. In *Fig. 9a *an entire femur is shown (arrow: Bone-fixation device), whereas in *Fig.9b *only the original defect with a rim of adjacent bone is pictured.

**Figure 10:**
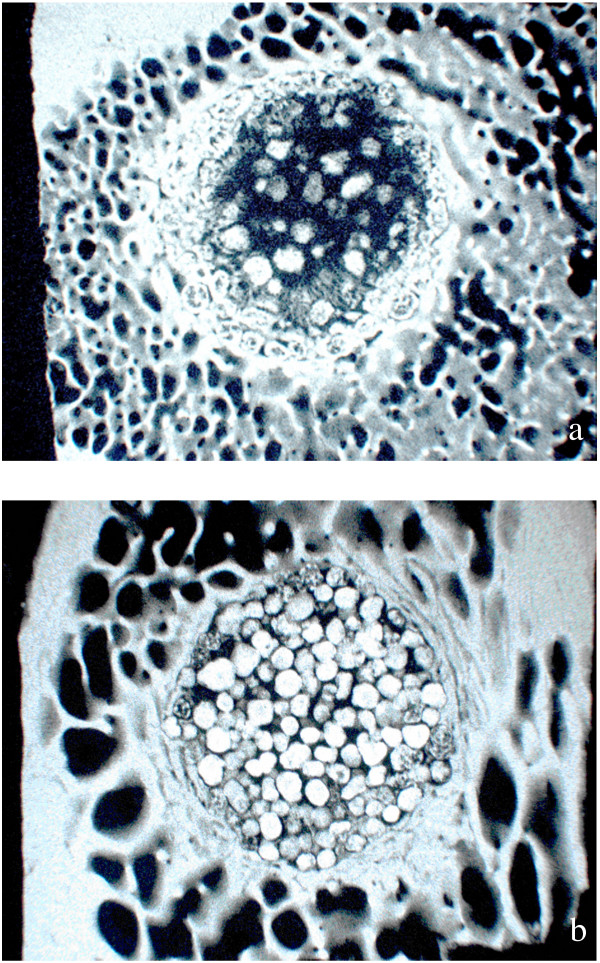
Microradiographs taken from two different biomaterial samples: Microradiographs show the stage of calcification of the bone after the application of bone enhancing materials (Fig. 10a) or both, remnants of bone cements based on calcium phosphate and replacement by new bone (Fig. 10b).

After the sheep were sacrificed both humeral and femoral bones were harvested immediately, freed from all overlying soft tissues and, if possible, the drill hole in the lateral cortex was identified. Time points of sacrifice varied from 2, 4, 8, 16 to 24 weeks depending on the original study question. The long bones were radiographed again using a faxitron machine (Cabinet x-ray-faxitron series, model 43855A, Hewlett Packard^®^, USA) that allows visualizing the detailed bone structure of the trabecular bone. Thereafter, bone blocks were cut using a bone saw (K 410, Kolbe GmbH, Elchingen, Germany) assuring a rim of at least 3–4 mm of adjacent bone at the side and at the bottom of the original bone defect. Care was taken that the blocks were sectioned parallel to the original bone cylinder (Fig. 8). If important in relation to the original study question (e.g. fine trabecular structure within bone defect or adjacent bone) the blocks were radiographed again with the faxitron machine (Fig. 9) or they were subjected to micro-computed tomography. All samples were fixed in either 4% paraformaldehyde or 10% fresh, buffered formaldehyde, before they proceeded for histology of non-decalcified bone section as described elsewhere [42,43]. Briefly, after fixation, samples were washed in buffered saline, dehydrated in a series of alcohol, defatted in xylene under vacuum, infiltrated in methylmethacrylate (methacrylacid-methylester; dibuthylphtalate and perkadox in a proportion 89,5: 10: 0,5) and then finally embedded in the same solution using special Teflon molds placed in a standard water bath at 30°C. Ground sections (30–40 *μ*m) and thin sections (5 *μ*m) were prepared using special equipment (Leica^® ^SP 1600 and Leica^® ^RM 2155; Leica Instruments GmbH, Nussloch, Germany). Sections were either surface stained with toluidine blue in case of ground sections, or deplastified with Methoxyethyl-acetate (Merck AG, Switzerland) and stained with either toluidine blue or von Kossa/McNeil in case of thin sections. Before mounting the ground sections to the opal, acrylic Plexiglas slides (Wachendorf, Perspex GS, Acrylicglas Opal 1013) microradiographs were taken to visualize the stage of calcification of the bone samples within and adjacent to the biomaterial (Fig. 10). Sections were cut in the middle of the bone defect and perpendicular to the original drill hole revealing the round original bone defect for further histological assessment with light microscopy. Ground sections were used to determine resorption of biomaterials, new bone formation and bone remodelling of the adjacent bone, but also to measure the area and percentage of bone, fibrous tissue and remnants of biomaterials within the original defect by means of histomorphometry (Leica Qwin^®^, Leica Quips^®^, Leica, Glattbrugg, Switzerland). For this, pictures were captured with the macroscope as digital images in TIF-format (DC 200, Leica. Glattbrugg, Switzerland) and at a 5.8 times magnification. Thin sections were suitable for semi-quantitative evaluation of samples with the light microscope (Leica, DMR, Glattbrugg, Switzerland), where cellular reactions were assessed based on a semi-quantitative score that was specifically developed for each individual study [43].

## Results

Anaesthesia and recovery phases were uneventful for 51 of the 55 animals. As soon as the sheep were awake they were placed in straw-lined small stalls. Ten days later, after removal of the skin staples, animals were released to larger stalls and finally were allowed to roam free on pasture after 20 days. At no time did they show signs of lameness or other discomfort.

For 4 sheep, the positioning of the drill holes was not exactly in the correct places described above, but slightly off towards the diaphysis of the femur or distal humerus respectively. These animals suffered either from a comminuted fractured humerus (n = 1) or femur (n = 3) within the first 3 postoperative days. All four animals were immediately euthanized after the complications occurred. All other drill holes (total of n = 528 holes) could be easily placed except in the distal humerus, where in 9 instances the drill hole was initiated too far cranially and distally resulting in slippage of the drill bit into the cranial pouch of the elbow joint including an incomplete drill hole within the cranial aspect of the lateral humeral condyle. In those 9 instances, where initial slippage of the drill bit occurred from the lateral humeral condylus, the surgeon gave up drilling the hole to avoid spontaneous fractures of the distal humerus. There, the biomaterials were never applied.

After the pre-planned time period the animals were slaughtered and the treated bones removed. Depending on the time point of sacrifice the original drill hole was easily detected or especially at later time points almost healed, at least at the cortical site. But even then, there was a small amount of connective tissue visible in the area indicating the original defect within the cortical bone. No macroscopic signs of inflammatory reactions were generally seen even at the early time points. However, this was dependent on the implanted material [44]. Normally, the radiographs taken with the faxitron machine revealed the location and direction of the drill hole, although at later time points (24 weeks) depending on the biomaterials bone healing could be so far advanced that detection proved to be difficult and only signs of intensive bone remodelling indicated the original bone defects.

**Figure 11:**
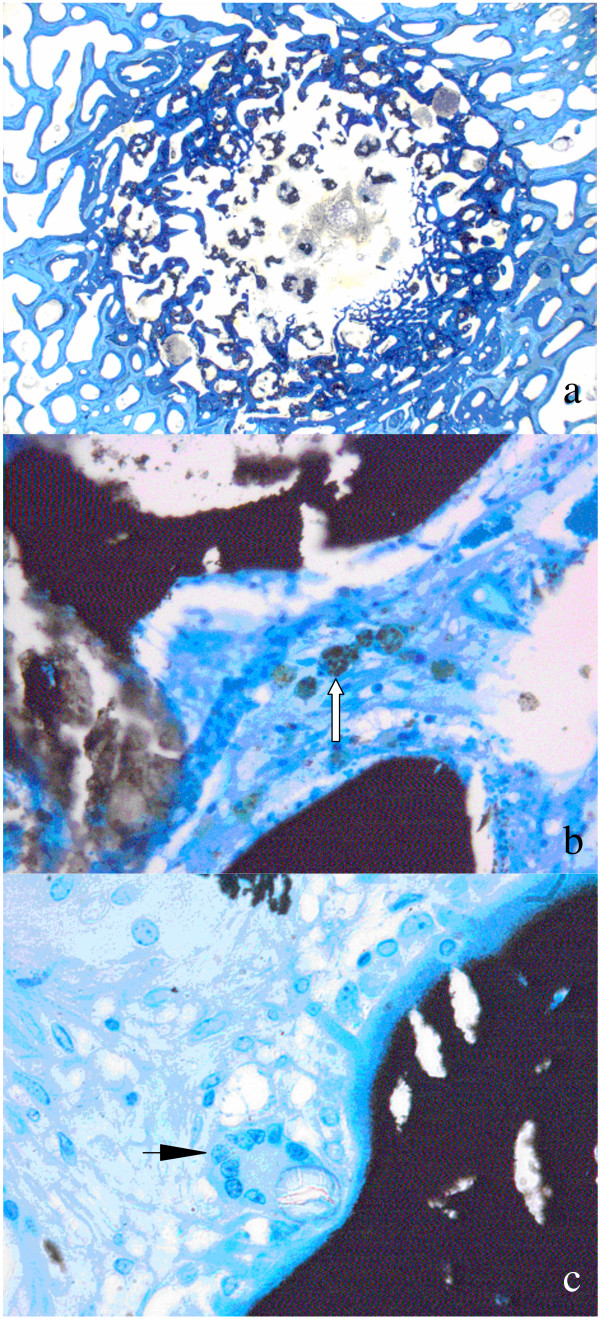
Histology samples: While ground sections (30–40 *μ*m, surface stained with toluidine blue) are well suited for the assessment of osseointegration, new bone formation, materials resorption and histomorphometrical measurement (Fig. 11a), thin sections allow assessing cellular reactions such as degradation and elimination of biomaterials through macrophages (Fig. 11b, arrow) or the appearance of foreign body cells (Fig. 11c, arrow).

The bone samples could be easily embedded in acrylic resin and in all instances the size of samples allowed the preparation of non-decalcified histology sections for ground and thin sections. Depending on the biomaterials and stage of osseointegration or resorption the sectioning of thin sections required great skill from our histology technician. This was especially true for sections containing calcium phosphate bone cements where the biomaterial was brittle and if no bone replacement had taken place yet in the early sections (2, 4 and 8 weeks) the material simply disintegrated during sectioning. However, this hardly occurred in the ground sections (30 – 40 *μ*m). There, the defect could be clearly distinguished from the surrounding bone. Thin sections allowed distinguishing cellular events in all instances even if the bulk of biomaterial was fallen off during sectioning since the immediately adjacent tissue could still always be evaluated (Fig. 11).

The time frame up to 6 months proved to be adequate for this size of defect, since most of the biomaterials tested were resorbed and replaced by new bone to at least two thirds of the original bone defect within this period. The same was true for the assessment of cellular events within this given time point. Degradation mechanisms could be easily followed at the interface between biomaterials and host tissue. The early time points (2, 4 weeks) were helpful to test biocompatibility issues since the appearance of foreign body or other mononuclear cells indicative of host reaction to the material occurred mainly within this early phase.

## Discussion

The drill hole model in sheep as presented is well suited to test biocompatibility issues regarding biodegradable materials, but also to answer questions related to material resorption, substitution with new bone or less functional tissue such as fibrous tissue. The experimental model was established in 70 sheep with a total of 560 drill holes for materials to be tested in bone. Several advantages are pertinent to this well standardized animal model with animal welfare being one of the most important aspects, closely followed by the possibility to translate results relatively easy to humans without the necessity of additional animal experiments related to biocompatibility questions.

The surgical technique requires a modified drill bit with a flattened tip, solid anatomical knowledge and experience of the surgeon. The small stab incisions and minimal preparation of soft tissues to access the bone cortex, where the drill hole will be placed, are part of the regimen of minimally invasive surgery and thus, atraumatic technique including prevention of unnecessary suffering of the animals.

**Figure 12:**
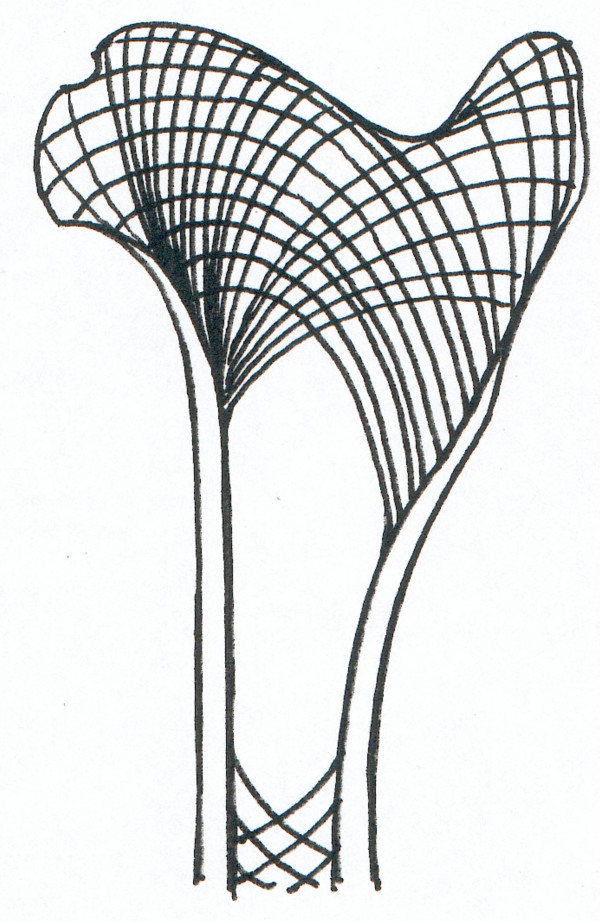
The graph represents a drawing of a longitudinal section of the proximal part of the femur. The lines picture the area where cancellous bone can be found, but also the trajectories indicating the direction of the mechanical tension lines in the bone.

The diligent and correct positioning of the limbs for surgery turned out to be a decisive factor for positioning the drill holes, especially for the distal humerus and femur. This allows optimal positioning of the drill hole that needs to be placed exactly as described in the surgical technique because of the quality of the brittle sheep bone. If the drill holes are slightly misplaced spontaneous fractures occur as described with the 4 animals with complications. The special structure and morphology of long bones in sheep corresponds to the function and mechanical load of the specific bone area [45]. This leads to a relatively large area of cancellous bone with a thin cortex in the metaphysis and epiphysis of the humerus and femur. Here, the mechanical pressure can be transferred to the cancellous network, while closer to the diaphysis this is not possible any longer since there is only cortical bone and fatty bone marrow to withstand the mechanical load (Fig. 12). The relative large drill hole of 8 mm in relation to the overall bone diameter obviously disturbs the tension lines of the bones significantly and gives rise to spiral fissures and cracks along these so called trajectories. This occurs even if only one cortex is drilled and is usually associated with severely comminuted fractures. If the drill holes are correctly placed within the metaphysis (proximal humerus and femur) or epiphysis (distal humerus and femur) this complication can be avoided. Especially in the distal humerus it may be better to stay away from drilling the hole if in doubt whether the position is correctly chosen. When the method was performed correctly, as described above, then there were no unforeseen fractures. In 9 cases the drill bit slipped into the cranial pouch of the elbow joint. To avoid spontaneous fractures, the drilling was not repeated and no biomaterial was applied. Since we only touched the joint but never penetrated any important structures, it had no clinical implication such as haematoma, pain or lameness.

**Figure 13:**
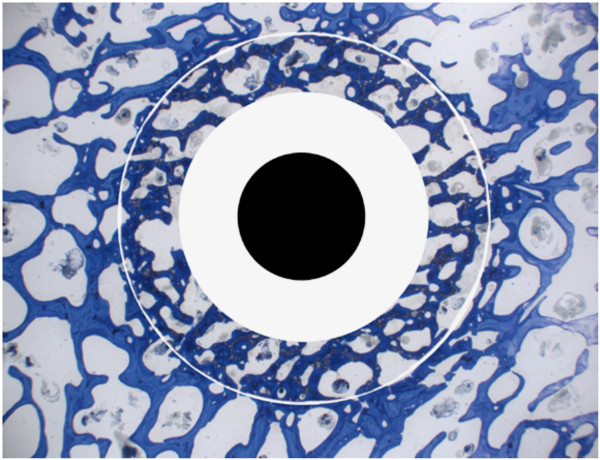
The picture shows the division in peripheral, middle and centre area of the bone defect as may be necessary to compare bone formation and biomaterials resorption over time or between different materials.

Histology with non-decalcified bone sections proved to be suitable to assess bone healing as well as the behaviour of biodegradable materials. By sectioning the bone samples in midlength and perpendicular to the original drill hole the stage and ratio of new bone formation versus material resorption could be optimally visualized and calculated at its most remote location. Since any biomaterial used in bone finally will be replaced by creeping substitution [46] it may be safely assumed that if the centre and middle of the original biomaterial is replaced with new bone the rest of the original bone defect has already been replaced and is already undergoing bone remodelling. If deemed necessary serial sections can be prepared throughout the entire original defect, although the extensive workload and costs associated with preparing sections of non-decalcified bone may prove inhibitive. The standardized drill hole defect is well suited for histomorphometrical measurements where an automatic software program with the appropriately established "macro" allows the calculation of area percentages of bone, remnants of biomaterials as well as replacement with fibrous tissue. Apart from the overall percentage of each component the area may be divided in rings facilitating the comparison of the peripheral, middle and more central areas of the original bone defect [43]. This is interesting especially if the rate of resorption over time or between different biomaterials should be compared (Fig. 13).

It is common belief that for ethical reasons small experimental animals, such as mice, rats or rabbits, should be used wherever possible. Another reason to turn to small rodents is for economical aspects as rats and mice are much less expensive and easier to maintain as sheep. Biocompatibility of novel materials is often tested in less demanding animal models in the literature. Biological materials implanted into soft tissue, for example intramuscularly [9,16,29] or subcutaneously [12,14,17,33]), may answer questions pertaining to the local cellular and humoral reaction, but not to the degree of new bone formation, osteoinduction and -conduction. Even the local cellular degradation mechanisms may be different in soft and osseous tissue, since osteoclasts often involved in synthetic hydroxyapatite-based bone materials, are only present in bone but not subcutaneous or muscular tissues. In addition, any material placed in soft tissue may elicit the formation of a soft tissue capsule in an attempt to wall it off. This reaction most likely has nothing in common with a response related to biocompatibility, but could also be a reaction to mechanical instability and different stiffness and rigidity of soft tissue and the implanted biomaterial. In the author's judgement the subcutaneous and intramuscular pouch model in rodents as introduced by Urist et al [47,48] is a valuable animal model for heterotopic osteoinduction triggered by biomimetic substances, but not for testing biocompatibility issues related to bone.

There are successful reports with drill holes used in rabbit tibias [23,26] and rat femurs [11]. Rodents may require less time and cost as well as less sophisticated surgery and anaesthesia facilities. However, more animals per study may be required due to limited anatomical space and size of animals. Furthermore, bone metabolism is significantly different with mostly faster bone remodelling. Positive results in rodents may have to be repeated and verified in larger species before human clinical trials can be initiated. Because of the similarities with human bone metabolism the results from sheep carry more authority than those obtained small laboratory animals [34].

The drill hole model as presented here has additional advantages. With this model it is possible to investigate and compare the in vivo characteristics of up to eight different materials in one animal. Also it is possible to compare more than one sample of the same material within an animal but in different locations representing different densities of cancellous bone. This is not just a question of animal protection, but also adds the possibility to compare directly the individual's reaction to various materials. Last but not least, this model allows testing of biomaterials without other than the physiologic mechanical load, which is in contrast to animal models that produced single injection or implantation sites using osteotomy or ostectomies [24,30,49]. These models may not be as suitable for biocompatibility testing of materials in bone as the drill hole model, since it may be difficult to attribute failures to material incompatibility or mechanical problems. Critical sized bone defects in long or cranial bones are the next step once the biocompatibility of the material has been established [31,32,50,51].

## Conclusion

The drill hole model in sheep proves to be an excellent animal model to test biocompatibility of biomaterials that should be implanted in bone. Overall, it is a safe model for the experienced surgeon, reduces suffering and numbers of experimental animals and serves as an excellent basis for testing the best materials in a more challenging animal model where mechanical properties play a role such as in critical sized bone defects of long, maxillofacial or cranial bone.

## Competing interests

The author(s) declare that they have no competing interests.

## Authors' contributions

KN participated in the design of the study, carried out a part of the surgeries, participated in the evaluation of the sections and drafted the manuscript.

JA participated in the design of the study and carried out a part of the surgeries.

AB is responsible for the anatomical part of the study.

BvR participated in the design of the study, carried out a part of the surgeries, supervised the projects and has been involved in drafting and revising the manuscript.

All authors read and approved the final manuscript.

## Pre-publication history

The pre-publication history for this paper can be accessed here:



## References

[B1] Korner D, Palluck E (1991). Tierexperimentelle Untersuchungen zur Biokompatibilitat von Wurzelfüllmaterialien. Dtsch Z Mund Kiefer Gesichtschir.

[B2] Ramakrishna S, Huang Z, Kumar G, Batchelor A, Mayer J (2004). An introduction to biocomposites.

[B3] Lane J (1987). Current approaches to experimental bone grafting. J orthop clin north am.

[B4] Hing KA, Best SM, Tanner KE, Revell PA, Bonfield W (1998). Histomorphological and biomechanical characterization of calcium phosphates in the osseous environment. Proc Inst Mech Eng [H].

[B5] Bauer T (2000). Bone graft materials. An overview of the basic science. J clin orthop.

[B6] Williams DF, de Putter C, K dL, de Groot K, Lee A (1988). Consensus and definitions in biomaterials. Advances in biomaterials.

[B7] Ratner B (1993). New ideas in biomaterials science- a path to engineered biomaterials. J Biomed Mat Res.

[B8] Hunziker EB (2003). Tissue engineering of bone and cartilage. From the preclinical model to the patient. Novartis Found Symp.

[B9] Endres S, Landgraff M, Kratz M, Wilke A (2004). Biokompatibilitätstestung verschiedener Biomaterialien in Abhängigkeit vom Immunstatus. Z Orthop Ihre Grenzgeb.

[B10] Ellies LG, Carter JM, Natiella JR, Featherstone JD, Nelson DG (1988). Quantitative analysis of early in vivo tissue response to synthetic apatite implants. J Biomed Mater Res.

[B11] Thull R, Trautner K, Karle EJ (1992). Modell zur immunologischen Prüfung von Biomaterialien. Biomed Tech (Berl).

[B12] Benghuzzi H (1996). Cytological evaluation of capsular tissue surrounding TCPL implant in adult rats. Biomed Sci lnstrum.

[B13] Butler K, Benghuzzi H, Bajpai P, Puckett A, Tucci M, Cason Z, England B (1997). One year histopathological evaluation of fibrous tissue surrounding TCPL implants using adult rats as a model. Biomed Sci lnstrum.

[B14] Held M, Dost P, Erdtmann M, Baumann H, Jahnke K (2000). Biocompatibility testing of new polymers in a moving implant bed. ORL J Otorhinolaryngol Relat Spec.

[B15] Fini M, Giavaresi G, Torricelli P, Krajewski A, Ravaglioli A, Belmonte MM, Biagini G, Giardino R (2001). Biocompatibility and osseointegration in osteoporotic bone. J Bone Joint Surg Br.

[B16] Mi FL, Tan YC, Liang HF, Sung HW (2002). In vivo biocompatibility and degradability of a novel injectable-chitosan-based implant. Biomaterials.

[B17] Fulzele SV, Satturwar PM, Dorle AK (2003). Study of the biodegradation and in vivo biocompatibility of novel biomaterials. Eur J Pharm Sci.

[B18] Lewandrowski KU, Bondre SP, Wise DL, Trantolo DJ (2003). Enhanced bioactivity of a poly(propylene fumarate) bone graft substitute by augmentation with nano- hydroxyapatite. Biomed Mater Eng.

[B19] Sousa CJ, Loyola AM, Versiani MA, Biffi JC, Oliveira RP, Pascon EA (2004). A comparative histological evaluation of the biocompatibility of materials used in apical surgery. Int Endod J.

[B20] Jinno T, Goldberg VM, Davy D, Stevenson S (1998). Osseointegration of surface-blasted implants made of titanium alloy and cobalt-chromium alloy in a rabbit intramedullary model. J Biomed Mater Res.

[B21] Lopes MA, Santos JD, Monteiro FJ, Ohtsuki C, Osaka A, Kaneko S, Inoue H (2001). Push- out testing and histological evaluation of glass reinforced hydroxyapatite composites implanted in the tibia of rabbits. J Biomed Mater Res.

[B22] Gao J, Knaack D, Goldberg VM, Caplan AI (2004). Osteochondral defect repair by demineralized cortical bone matrix. Clin Orthop Relat Res.

[B23] Orsini G, Ricci J, Scarano A, Pecora G, Petrone G, Iezzi G, Piattelli A (2004). Bone-defect healing with calcium-sulfate particles and cement: an experimental study in rabbit. J Biomed Mater Res B Appl Biomater.

[B24] Patka P, den Otter G, de Groot K, Driessen AA (1985). Reconstruction of large bone defects with calcium phosphate ceramics – an experimental study. Neth J Surg.

[B25] Choueka J, Charvet JL, Koval KJ, Alexander H, James KS, Hooper KA, Kohn J (1996). Canine bone response to tyrosine-derived polycarbonates and poly(L-lactic acid). J Biomed Mater Res.

[B26] Pal AK, Pal TK, Mukherjee K, Pal S (1997). Animal experimentation with tooth derived calcium hydroxyapatite based composites as bone-graft substitute biomaterials. Biomed Sci Instrum.

[B27] Bumgardner JD, Boring JG, Cooper RC, Gao C, Givaruangsawat S, Gilbert JA, Misch CM, Steflik DE (2000). Preliminary evaluation of a new dental implant design in canine models. Implant Dent.

[B28] Lee TM, Yang CY, Chang E, Tsai RS (2004). Comparison of plasma-sprayed hydroxyapatite coatings and zirconia-reinforced hydroxyapatite composite coatings: in vivo study. J Biomed Mater Res A.

[B29] Mendes SC, Reis RL, Bovell YP, Cunha AM, van Blitterswijk CA, de Bruijn JD (2001). Biocompatibility testing of novel starch-based materials with potential application in orthopaedic surgery: a preliminary study. Biomaterials.

[B30] Ignatius AA, Betz O, Augat P, Claes LE (2001). In vivo investigations on composites made of resorbable ceramics and poly(lactide) used as bone graft substitutes. J Biomed Mater Res.

[B31] Kümmerle J (2003). Experimentelle Studie über die Eignung eines neuen Brushite Calciumphosphatzementes für die kraniofasziale Knochenchirurgie anhand eines ovinen Modells der parietalen Kranioplastik und maxillären Augmentation. Doctoral Thesis.

[B32] Theiss F, Apelt D, Brand B, Kutter A, Zlinsky K, Bohner M, Matter S, Frei C, Auer J, von Rechenberg B (2005). Biocompatibility and resorption of a brushite calcium phosphate cement. Biomaterials.

[B33] Bush D, England B, Tucci M, Cason Z, Lemos L, Benghuzzi H (1995). The effect of TCPL devices on tissue-implant interface analysis using adult sheep as a model. Biomed Sci Instrum.

[B34] Nunamaker D (1989). Experimental models of fracture repair. J Clin Orthop Rel Res.

[B35] Augat P (1998). Local tissue properties in bone healing: Influence of size and stability of the osteotomy gap. J Orthopaed Res.

[B36] Sarkar M, Patka P, Kinzl L (2001). First histological observations on the incorporation of a novel calcium phosphate bone substitute material in human cancellous bone. J Biomed Mat Res.

[B37] Bosanquet A, Goss A (1987). The sheep as a model for temporomandibular joint surgery. Int J Oral Maxillofac Surg.

[B38] Arvier J, Scott J, Goss A, Wilson D, Tideman H (1989). Biological and clinical evaluation of the transmandibular implant. Aust Dent J.

[B39] Rice J, Cowin S, Bowman J (1988). On the dependence of the elasticity and strength of cancellous bone on apparent density. J Biomech.

[B40] Kuhn J, Goldstein SA, Ciarelli M, Mathews L (1989). The limitations of canine trabecular bone as a model for human: a biochemical study. J Biomech.

[B41] Kemper M (2003). Screeningversuch zur Optimierung synthetischer und fibriner Hydrogele als dreidimensionale Trägersubstanz für Bone Morphogenetic Protein, Transforming Growth Factor-beta und Parathormon zur Beschleunigung der Knochenheilung. Doctoral Thesis.

[B42] Apelt D, Theiss F, El-Warrak A, Zlinski K, Bettschart-Wolfensberger R, Bohner M, Matter S, Auer J, von Rechenberg B (2004). In vivo behaviour of three different injectable hydraulic calcium phosphate cements. Biomaterials.

[B43] Doernberg Cv (2005). Evaluation of the biodegradation behaviour of b-tcp cylinders with four different pore diameters. Doctoral Thesis.

[B44] Oberle A, Theiss F, Bohner M, Müller J, Kästner SB, Boecken I, Zlinsky K, Wunderlin S, Auer J, von Rechenberg B (2005). Untersuchungen über den klinischen Einsatz von Brushite- und Hydroxyapatite-Zement beim Schaf. Schweiz Arch Tierh.

[B45] Wolff J (1901). Über die Wechselbeziehungen zwischen der Form und der Funktion der einzelnen Gebilde des Organismus.

[B46] Phemister DB (1914). The fate of transplanted bone and regenerative power of its various constituents. Surg Gynecol Obstet.

[B47] Urist MR, Dowell TA (1968). Inductive Substratum for Osteogenesis in Pellets of Particulate Bone Matrix. Clin Orthop Relat Res.

[B48] Urist MR, Silverman BF, Buring K, Dubuc FL, Rosenberg JA (1967). The bone induction principle. Clin Orthop Relat Res.

[B49] Ignatius AA, Unterricker K, Wenger K, Richter M, Claes L (1997). A new composite made of polyurethane and glass ceramic in a loaded implant model: A biomechanical and histological analysis. J Mater Sci Mater Med.

[B50] Meinel L, Hofmann S, Karageorgiou V, Kirker-Head C, McCool J, Gronowicz G, Zichner L, Langer R, Vunjak-Novakovic G, Kaplan DL (2005). The inflammatory responses to silk films in vitro and in vivo. Biomaterials.

[B51] Meinel L, Zoidis E, Zapf J, Hass P, Hottiger MO, Auer J, Schneider R, Luginbuhl V, Bettschart-Wolfensberger R, Rechenberg Bv (2003). Localized insulin-like growth factor I delivery to enhance new bone formation. Bone.

